# Age-Related Macular Degeneration and Mitochondria-Associated Autoantibodies: A Review of the Specific Pathogenesis and Therapeutic Strategies

**DOI:** 10.3390/ijms25031624

**Published:** 2024-01-28

**Authors:** Sichang Qu, Hao Lin, Norbert Pfeiffer, Franz H. Grus

**Affiliations:** Department of Experimental and Translational Ophthalmology, University Medical Center, Johannes Gutenberg University, 55131 Mainz, Germany; sicqu@uni-mainz.de (S.Q.); haolin@uni-mainz.de (H.L.);

**Keywords:** age-related macular degeneration, retinal pigment epithelium, autoantibodies, mitochondria, therapeutic strategies

## Abstract

Age-related macular degeneration (AMD) is a severe retinal disease that causes irreversible visual loss and blindness in elderly populations worldwide. The pathological mechanism of AMD is complex, involving the interactions of multiple environmental and genetic factors. A poor understanding of the disease leads to limited treatment options and few effective prevention methods. The discovery of autoantibodies in AMD patients provides an opportunity to explore the pathogenesis and treatment direction of the disease. This review focuses on the mitochondria-associated autoantibodies and summarizes the functional roles of mitochondria under physiological conditions and their alterations during the pathological states. Additionally, it discusses the crosstalk between mitochondria and other organelles, as well as the mitochondria-related therapeutic strategies in AMD.

## 1. Introduction

### 1.1. Overview of AMD

Age-related macular degeneration (AMD) is the leading cause of progressive and irreversible blindness in people over 60 years of age in Western countries. According to an epidemiological survey, the number of patients worldwide is estimated to reach 288 million in 2040 [1]. In addition to impaired visual function in daily activities, AMD can lead to depression, anxiety, and adjustment disorders, which places a considerable socioeconomic burden on healthcare systems [2].

**Figure 1:**
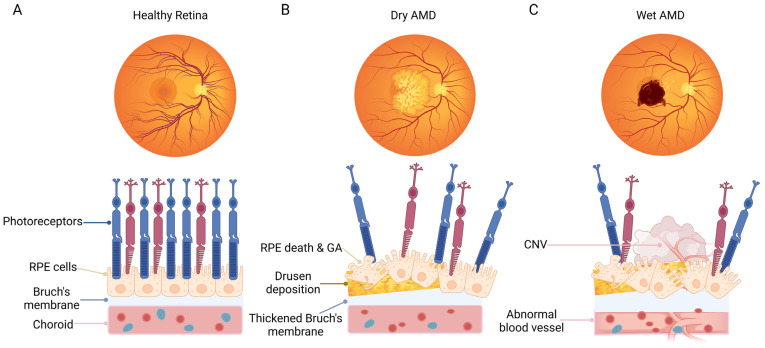
Schematic representation of fundus and macular cell organization in normal subjects and patients with dry AMD or wet AMD. (**A**) Healthy fundus with a normal cellular structure of the macular area. (**B**) A representative eye with dry AMD, showing dead or dying photoreceptors and RPE cells, geographic atrophy, drusen deposition, and a thickened Bruch’s membrane. (**C**) A representative eye with wet AMD, showing dead or dying photoreceptors, RPE cells, as well as the formation of choroidal neovascularization. The Bruch’s membrane is damaged, accompanied by neovascularization in the choroid plexus. The figure was created using BioRender.com (https://biorender.com/, accessed on 20 December 2023).

Conventionally, AMD is divided into two main forms, including wet AMD and dry AMD (Figure 1). Dry AMD is manifested by drusen, pigmentary abnormalities, and geographic atrophy (GA) [3]. Wet AMD is characterized by the formation of choroidal neovascularization (CNV), the presence of subretinal fluid or hemorrhage, retinal pigment epithelial (RPE) detachment, and scar fibrosis [4]. Clinically, the rapid visual loss in patients with wet AMD is usually caused by the pathological CNV-induced hemorrhagic detachment of the retina. In addition, drusen deposition and GA in the advanced stage of the dry form can lead to the irreversible loss of retinal cells, eventually resulting in the loss of visual function. The most serious risk factor for AMD is aging [5]. Furthermore, multiple factors have also been shown to be correlated with AMD, including environmental factors (e.g., smoking), low-density lipoprotein pathway-related factors (e.g., apolipoprotein (APO) E), obesity, cardiovascular-related risk factors, as well as genetic polymorphisms factors related to the complement pathway, e.g., complement factor H, complement factor B, and complement components 2 and 3 [6,7,8]. The pathological mechanism of AMD is complex, including oxidative stress, inflammation, and complement system activation, the dysregulation of lipid metabolism, angiogenesis, and extracellular matrix remodeling [9,10,11,12].

In clinical practice, the intravitreal injection of anti-vascular endothelial growth factor (VEGF) has become the standard therapy for wet AMD. The administration of ranibizumab, bevacizumab, or aflibercept alleviates the condition to a great extent. However, it should not be ignored that many patients respond inadequately or the treatment loses efficacy with the repeated administration of anti-VEGF drugs [13]. For dry AMD, several novel methods, such as complement inhibition, visual cycle modulators, neuroprotection, cell-based therapies, and gene therapy, have shown their potential [14]. However, there are still no revolutionary advances in treatment. Therefore, it is particularly important to find effective and targeted treatments for the disease.

### 1.2. Functions of RPE Cells

The most crucial target of AMD pathophysiology is RPE cells. It is a monolayer of cells located between the neural retina and the choroid, and the cells are highly polarized. On the apical side, their microvilli interact with photoreceptor outer segments to perform a phagocytotic function. On the basal side, they exchange oxygen, nutrients, and waste with Bruch’s membrane and the choroid capillaries. The RPE layer is essential to maintain the health and functionality of neural retina, including synthesizing and storing melanin; keeping the volume, ion concentrations, and chemical composition of the sub-retinal space; participating in the visual cycle; and regulating retinal immune responses [15,16,17]. The specific location and high metabolic activity of RPE cells lead to their increased susceptibility to oxidative stress and accumulated damage. With aging, RPE cells undergo cellular senescence and have a reduced repair capacity, superimposed by persistent environmental factors, which ultimately lead to cell death [18,19].

This review takes RPE cells as the main object and explores the pathogenesis and potential treatments of AMD from the perspective of autoantibodies and mitochondria.

## 2. Autoimmune Processes in AMD

The eye is an immune-privileged site that protects the retinal tissue from inflammatory damage, but this status may leave the eye vulnerable to autoimmune attacks [20]. In the context of the pathogenesis of AMD, there is an ample evidence of autoimmunity involvement, and some also propose that AMD may be an autoimmune disease [21,22,23]. Autoantibodies are weapons that target the body’s own tissues or molecules. During AMD, they mistakenly target specific components of the retina, such as the RPE. Multiple autoantibodies are found in patients with different types and stages of AMD, and the IgG immunoreactivity of these antibodies are both significantly upregulated or downregulated. A study observed elevated levels of retinal autoantibodies against 28–49 kDa retinal proteins in 94% of patients with early AMD and 83% of patients with advanced AMD, compared to 9% of normal controls [24]. In AMD, autoantibodies play a role in inflammation and immune responses. Some of these autoantibodies are related to mitochondrial functions to a certain extent [24]. In this paper, we listed some of the potential mitochondria-related autoantibodies in AMD patients (Table 1) and explained the effects of changes in mitochondrial function on AMD in the following sections.

The presence of autoantibodies against glial fibrillary acid proteins (GFAPs) in the serum of AMD patients was first reported in 1990 and also confirmed in our study [21,26]. It is an established marker of retinal astrocytes. According to a study, the upregulation of GFAP in the inner retina was associated with drusen formation and upregulation in the outer retina was associated with the disruption of the RPE and blood–retinal barrier [21]. In an ethanol-induced study of rat hippocampal astrocytes, the increased content of GFAP was shown to be associated with reactive oxygen species (ROS) generation and Ca^2+^ release [27]. Furthermore, it was shown that mutations in the GFAP gene disrupted the transfer of mitochondria from astrocytes to neurons [28].

According to our previous study, autoantibodies against alpha-enolase (α-enolase) were found to be upregulated in 67% of AMD patients. α-enolase is a glycolytic enzyme, which belongs to the heat shock protein family [29]. Autoantibodies against α-enolase are associated with retinal degeneration and visual loss in patients with autoimmune retinopathy [30]. In a study, the application of Enol-1 monoclonal antibody (an antibody against alpha-enolase) significantly increased intracellular Ca^2+^ and inhibited the catalytic function of enolase, which resulted in the depletion of adenosine triphosphate (ATP). In addition, mitochondria-located α-enolase has been found to be beneficial in stabilizing the mitochondrial membrane [31].

In our clinical study, one of the significantly altered reactivities was that of antibodies against alpha-synuclein (α-syn) [25,26]. α-syn is a protein that can interact with mitochondria by binding to the outer mitochondrial membrane and can be imported under certain conditions [32]. Recent evidence links α-syn to mitochondrial dysfunction in neurodegenerative diseases. A study noted that it can affect visual manifestations in Parkinson’s disease by regulating retinal iron homeostasis [33]. In addition, the different degrees of immunoreactivity to α-syn in patients with dry and wet AMD may indicate different stages of disease progression.

Autoantibodies against annexin V were found to be upregulated in the serum of patients ranging from early to advanced AMD [26]. Annexin V is a phospholipid-binding protein and critical to retinal physiology [34]. This intracellular protein was confirmed to interact with 27 kDa mitochondrial polypeptides and promote the transport of nuclear gene-encoded proteins to the mitochondria. Additionally, the Ca^2+^-binding properties of annexin V helps to combat the high levels of Ca^2+^ in the mitochondria via binding extra Ca^2+^ [34].

## 3. Mitochondria-Related RPE Physiology and AMD Pathology

**Figure 2:**
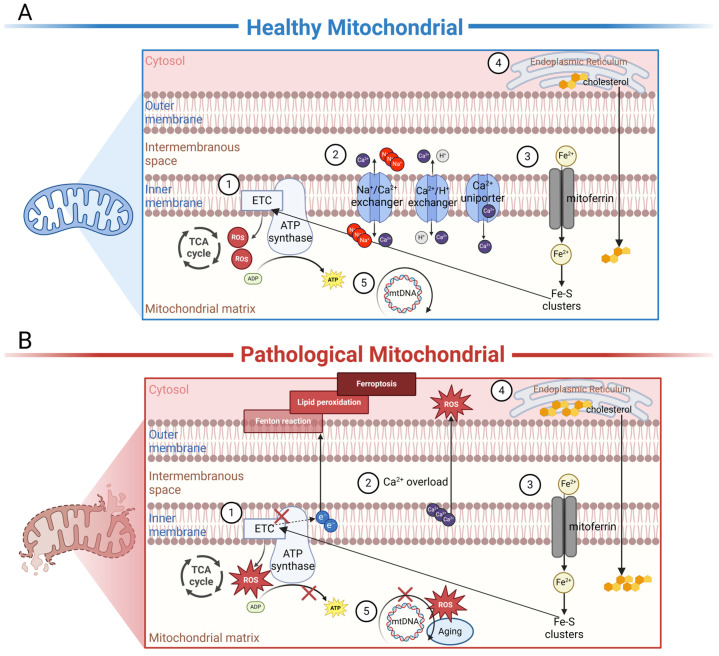
Schematic representation of the internal biochemical activities of mitochondria under healthy or diseased conditions. (**A**) In healthy mitochondria, ①. electron carriers produced by TCA enzymes donate electrons to the electron transport chain (ETC), driving the phosphorylation of ADP to ATP to meet the energy needs of the cell. ROS are important by-products of this process, and physiological concentrations of ROS are necessary for intracellular signaling. ②. Mitochondria are essential Ca^2+^-buffering organelles. Ca^2+^ flows between mitochondria and the cytoplasm via Ca^2+^ uniporter and the Na^+^/Ca^2+^ exchanger or the H^+^/Ca^2+^ exchanger. ③. Fe^2+^ is transported into mitochondria through mitoferrin; as an important component of the Fe-S cluster of the ETC, Fe^2+^ affects ATP production in mitochondria. ④. Cholesterol is originally synthesized in the ER and subsequently transported to the mitochondria for processing. ⑤. MtDNA encodes a series of polypeptides crucial for mitochondrial respiration and ATP production. (**B**) In dry or wet AMD, ①. excessive ROS can disrupt mtETC and affect ATP production. At the same time, tiny malfunctions in mtETC trigger a large increase in ROS production. ②. Under disease conditions, Ca^2+^ becomes a negative effector that damages mitochondria. An overload of Ca^2+^ is considerably released from damaged mitochondria. In cells, sustained high concentrations of Ca^2+^ can produce a large amount of ROS, leading to cell apoptosis. ③. Under disease states, electron leakage from ETC can cause the Fenton reaction, leading to the generation of lipid peroxides, thereby inducing cellular ferroptosis. ④. Cholesterol accumulation in mitochondria is involved in disease progression. ⑤. Excessive ROS can damage mtDNA-encoding genes and lead to the synthesis of functionally defective proteins. These proteins may further misdirect ROS overproduction, creating a vicious cycle. In addition, aging also causes spontaneous mutations in mitochondrial DNA. The figure was created using BioRender.com (https://biorender.com/, accessed on 24 January 2024).

Autoantibodies against mitochondria are widespread in multisystem diseases. This reminds us of the important role of mitochondria in the pathogenesis of these diseases, and the potential for therapeutics targeting mitochondria. In AMD, changes in RPE cell function at various stages were shown to be closely associated with decreased mitochondrial function. Accumulating evidence indicates that mitochondria in the RPE of AMD patients are damaged. By means of high-resolution imaging and 3D reconstruction, increased fragmentation as well as a decreased number and decreased integrity of mitochondria were observed in AMD patients compared to unaffected controls [35]. In addition, mitochondrial deoxyribonucleic acid (DNA) lesions were also found to be increased in the RPE cells of AMD patients, and the number of lesions increased with disease severity [36]. Therefore, we compiled an overview of mitochondrial functions in both healthy (Figure 2A) and diseased states (Figure 2B), aiming to explain the possible involvement of autoantibodies against mitochondria in AMD.

### 3.1. The Function of Mitochondria in the Normal RPE

Mitochondria are double-membrane organelles found in most eukaryotic cells. They contain separate and functionally distinct outer and inner membranes encapsulating the intermembrane space (IMS), cristae space, and matrix compartments [37]. The most notable function of mitochondria is the energy supply and the establishment of the membrane potential, which plays an important role in central metabolism. ATP is primarily generated by oxidative phosphorylation (OxPhos). In the matrix, electron carriers generated by tricarboxylic acid cycle (TCA) enzymes donate electrons to the electron transport chain (ETC). Complexes I to IV in the ETC pump protons from the matrix into the IMS through sequential conformational changes, which then drive the phosphorylation of ADP to ATP [38]. The number of mitochondria in each cell is determined by the energy demand [39]. Due to the high metabolic activity of retina, mitochondria are highly numerous in RPE cells, which meet their own energy needs and provide energy to nourish the photoreceptors (PR) [40]. Unlike other types of retinal cells, RPE cells can also utilize fatty acids to produce β-hydroxybutyrate as an alternative energy source with the help of mitochondria [41].

In addition to ATP production, mitochondria perform a variety of vital functions necessary to maintain cellular homeostasis. When mitochondria generate ATP, ROS are important by-products of this process. Under normal physiological conditions, 90% of ROS originate from mitochondria [42]. Although ROS have long been recognized as a main cause of RPE cell damage, low concentrations of ROS play an essential role in intracellular signaling, e.g., as secondary messengers that regulate cellular activities. As an important signaling molecule, ROS can transmit changes in the mitochondrial environment to the cytosol and nucleus via retrograde or anterograde signaling. Then, the cells can adapt to changing demands by modulating gene expression in the nucleus and mitochondria.

Calcium (Ca^2+^) is an important ion that initiates a series of biochemical reactions in RPE cells. Ca^2+^ homeostasis is required for cellular activities, including energy supply, cell signaling, the phagocytosis of photoreceptor outer segments, and the maintenance of cell polarization. The main role of mitochondrial Ca^2+^ is to synthesize ATP. An elevated Ca^2+^ concentration in mitochondria is coordinated by faster respiratory chain activity and a higher ATP output [43]. In addition, mitochondria are essential Ca^2+^-buffering organelles. Ca^2+^ enters mitochondria through the mitochondrial Ca^2+^ uniporter and returns to the cytoplasm by the Na^+^/Ca^2+^ exchanger or the H^+^/Ca^2+^ exchanger [44,45,46]. The Ca^2+^-loading capacity of isolated mitochondria is enormous, with a buffering capacity of approximately 10,000:1, which plays an important role in the regulation of Ca^2+^ signaling [47]. Furthermore, Ca^2+^ channels are also critical for signaling pathways, helping the communication between RPE cells and photoreceptors [48].

Another indispensable ion for the RPE is iron. It is critical for ATP production in mitochondria as an important component of cytochromes, cytochrome oxidase, and the iron–sulfur (Fe-S) clusters of the electron transport chain [49]. The uptake, import, and storage of iron rely on transferrin (TF), transferrin receptor (TFR), and ferritin, which are found to be expressed in RPE cells [50,51]. Transferrin receptors of human RPE cells, located on both the apical and basolateral sides, suggest a possible bidirectional flow of iron through the RPE [52]. The Fe^3+^ that enters cells is first converted to Fe^2+^ by metal reductase in the endosomes. Subsequently, the majority of the reduced Fe^2+^ is transported into the mitochondria via mitoferrins, where it is processed to metabolically active iron [50]. The intracellular iron homeostasis of RPE cells is considered to be independent of systemic regulation, because the related proteins are synthesized locally in the retina [51].

Although mitochondria are considered cholesterol-poor organelles, mitochondrial cholesterol has been shown to have vital physiological functions. Mitochondrial cholesterol levels are closely related to changes in antioxidant levels and OxPhos, and cholesterol metabolism in cells relies on the proper function of mitochondria [53]. Cholesterol is originally synthesized in the endoplasmic reticulum (ER) through the mevalonate pathway and subsequently delivered to the mitochondria via carrier-mediated and protein-mediated transport mechanisms across membrane contact sites [54,55]. The human RPE expresses all of the known components of the intracellular cholesterol transport, e.g., T trans locator protein, and cholesterol efflux components, like liver-X-receptor-α/β, ATP-binding cassette transporters (TSPO), APOA1, APOB, and APOE [56,57]. Cholesterol levels affect the fluidity, permeability, and various biophysical properties of the mitochondrial membrane, and fluctuations in these levels lead to changes in mitochondrial function.

The mitochondrial DNA (mtDNA) is a circular double-stranded molecule of 16,569 base pairs, which is localized in the inner matrix [58]. The DNA encodes a series of polypeptides crucial for mitochondrial respiration and ATP generation, including 13 subunits of complexes I, III, IV, and V [19,59]. MtDNA encodes ribonucleic acids (RNAs) for mitochondrial translation, including 22 transfer RNAs and 2 ribosomal RNAs (12S and 16S rRNA) [58]. Unlike nuclear DNA, which is protected by histones, mtDNA lacks structural protection and efficient repair mechanisms, making it particularly vulnerable to certain stress-induced damage [60,61].

Under normal conditions, dysfunctional mitochondria can be eliminated by mitophagy, a form of selective macroautophagy that primarily targets mitochondria [62]. Mitophagy acts as a quality control mechanism and is an essential component in maintaining cellular homeostasis when cells are faced with adverse conditions [63]. If this self-clearing work cannot be completed in time, the damaged mitochondria could also be the source of ROS, cytochrome c, and other apoptosis-related factors, which may lead to cell damage or even cell death [64]. Studies have proved that nutraceuticals or drugs that promote mitophagy may combat mitochondrial dysfunction, leading to the progression of dry AMD [65]. In addition, the metabolic re-programming of RPE cells in early AMD can be regulated by affecting mitophagy [66].

In contrast to the removal of damaged mitochondria by mitophagy, mitochondrial biogenesis is the way in which new mitochondria are produced and mitochondrial enzymes are turned over. It is the process of mitochondrial self-replication, which is orchestrated by mtDNA replication, transcription, and translation, as well as nuclear transcription [67]. Impaired mitochondrial biogenesis has been reported, in human RPE cells, as being affected by AMD [68]. The master regulators of mitochondrial biogenesis are peroxisome proliferator-activated receptor γ coactivator (PGC)1-α and nuclear respiratory factors (NRFs) [69]. Studies have shown that the activation of PGC1-α can slow down mitochondrial senescence and repress the epithelial–mesenchymal transition progression of RPE cells through mitochondrial biogenesis, while the inhibition of mitochondrial biogenesis by knocking down specific genes can induce mitochondrial-dependent apoptosis in RPE cells [70,71].

### 3.2. The Mitochondria-Related Pathogenesis in AMD

It is widely accepted that oxidative stress plays an important role in the development of AMD. In a two-level model hypothesis for AMD pathology, oxidative stress is considered the first level of molecular damage that marks a second-level oxidative burst and inflammation [72]. ROS are the executors of oxidative stress, which encompass a wide variety of chemical species, including hydrogen peroxide (H_2_O_2_), superoxide anion radical (O_2_^−^), hydroxyl radicals (OH·), alkoxy radical (RO^·^), peroxyl radical (ROO^·^), and others [73]. The threat of oxidative stress depends on the intensity of ROS-induced damage and cellular response. Damage to tissues or cells occurs when antioxidant defenses fail to clear excessive ROS.

Of all retinal organelles, mitochondria are particularly sensitive to oxidative stress due to their physiological position and function. In RPE cells, mitochondria aggregate at the basal side of the cell, as close as possible to the choriocapillaris, and migrate to oxygen sources, making mitochondria more susceptible to environmental stress [74]. ROS are released as important by-products of mtETC. When stress ripples through mtETC functions, the tiny malfunctions of mtETC trigger a substantial increase in and accumulation of ROS. More importantly, as mtDNA is very sensitive to oxidative damage, the excessive ROS could damage mtDNA-encoding genes and lead to the synthesis of functionally deficient proteins. These proteins may further misdirect the overproduction of ROS, creating a “mitochondrial vicious cycle” and thereby affecting a range of cellular activities that contribute to the development of AMD [75]. In addition, ROS is also known as a trigger for mitochondrial permeability transition pore (mPTP) opening, which is typically regarded as a pathological event [76]. At higher ROS levels, longer MPTP openings may release bursts of ROS, leading to the destruction of mitochondria. When this damage propagates from one mitochondria to another, it causes damage to the cell itself [77].

As mentioned earlier, Ca^2+^ seems to be a global positive effector of mitochondrial function. However, it can switch from a positive effector to a negative one under pathological stimulus [43]. It is well established that mitochondrial Ca^2+^ overload in the mitochondria matrix induces the opening of the mPTP, allowing the redistribution of various ions and solutes [78]. At this point, the properties of the inner mitochondrial membrane (IMM) become permeable and less selective. Any IMM molecule exhibiting a gradient is likely to passively diffuse into the intermembrane space and be subsequently released into the cytoplasm through the semipermeable outer mitochondrial membrane. The ion-induced reverse redistribution of H_2_O can lead to mitochondrial swelling, while the sustained opening of mPTP results in the loss of mitochondrial membrane potential. As the substrate is continuously diluted, ATP synthase halts ATP synthesis, resulting in a rapid blockade of ATP-dependent reactions. In this case, mitochondria are unable to create the conditions required to turn off mPTP, leading to mitochondrial damage and ultimately even cell death [79]. Since mitochondria are also Ca^2+^-buffering pools, damaged mitochondria release a large amount of Ca^2+^. In cells, the sustained high concentrations of Ca^2+^ generate large numbers of ROS, resulting in enzyme activation, the destruction of organelle structures, and cell apoptosis [80]. In addition, a study showed that the Ca^2+^ inflow could trigger lipofuscin accumulation in RPE cells and thus promote the AMD process [81].

The role of iron in the progression of AMD has received increased attention in recent years. Studies have shown that iron levels in the aqueous humor and iron deposition in the pathological retina of AMD patients are higher than in controls [82,83]. The levels of iron homeostasis-related mRNAs and proteins, such as TF, are found to be increased in the serum and macular region of AMD patients [84,85,86]. In addition, polymorphisms in iron homeostasis genes, such as TFR1 and TFR2, have been shown to be associated with risk factors for AMD [87]. Excessive iron can be toxic and induce ferroptosis in RPE cells, contributing to the development of AMD [88]. The ferroptotic cells exhibit marked mitochondrial changes, including reduced mitochondrial cristae, altered lipid peroxidation density of the mitochondrial membrane, outer mitochondrial membrane rupture, and mitochondrial fragmentation, all of which suggest a potential role of mitochondria in ferroptosis [89]. However, the regulatory relationship between mitochondrial activity and ferroptosis is still unclear. It is well known that mitochondria are the main source of cellular ROS, during which electron leakage from ETC leads to Fenton reaction and then generates lipid peroxides [90]. Thus, it has been supported that mitochondrial ROS generation contributes to ferroptosis by promoting lipid peroxidation [91]. Furthermore, a study has shown that mitochondria-targeted nitroxide can inhibit ferroptosis, which also supports this view [92]. In contrast, mitochondrial iron metabolism has been shown to play a positive role in ferroptosis defense and a study showed that the overexpression of mitochondrial ferritin can inhibit erastin-induced ferroptosis by promoting iron storage in mitochondria [93]. Exactly what role mitochondria play in ferroptosis, and whether their role switches under physiological and pathological states, deserve further investigation.

Cholesterol dysregulation is another pathological mechanism of AMD. This theory was proposed when cholesterol was first found to accumulate in the Bruch’s membrane of aging people [94]. Subsequently, in the component analysis study of the characteristic pathological changes in AMD, it was found that all kinds of drusen contained cholesterol, and hard drusen in particular had a cholesterol content of more than 40% [95]. In addition, genome-wide association studies further confirmed that cholesterol-related genes can influence susceptibility to AMD [96]. The current evidence suggests that cholesterol accumulation in mitochondria may be an important step in disease progression. It has been shown that the excessive enrichment of cholesterol in the mitochondria of specific tissues can lead to mitochondrial dysfunction and impaired transporters [97]. A study showed that the expression level of the intracellular cholesterol transport protein TSPO was decreased in aged RPE cells, and this correlated with cholesterol accumulation [98]. Furthermore, the reduced expression of cholesterol transporters in RPE cells has also been shown to increase the levels of pro-inflammatory cholesterol in the retina [99].

Genetic alterations in mitochondria are critical to the etiology of AMD, and the extent of mtDNA damage in the RPE correlates with the degree of AMD. One of the main drivers toward the damage of mtDNA is aging. According to studies, an increased disruption of the mitochondrial structure and a decreased mtDNA repair capacity are positively correlated with age [100]. This results in a reduced ability to resist oxidative damage and/or exhibit anti-inflammatory effects. Aging can also lead to spontaneous mutations in mtDNA. A study showed that the retinas of AMD patients exhibited increased mtDNA single-nucleotide polymorphisms in the control region compared to normal retinas [101]. Furthermore, apart from the age-related accumulation of mtDNA mutations, the overlapping impact of environmental factors, such as pollutants, ultraviolet radiation, and pharmaceuticals, also plays an important role in increasing the frequency of mtDNA mutations [61,102]. When damage influences the mitochondrial genome-encoding ETC subunits, it can lead to reduced ATP production, which in turn affects various cellular activities. As the bearer of genetic information, damage and mutations in mtDNA contribute to the progression of AMD.

## 4. The Crosstalk between Mitochondria and Other Organelles

### 4.1. Mitochondria and the Endoplasmic Reticulum (ER)

**Figure 3:**
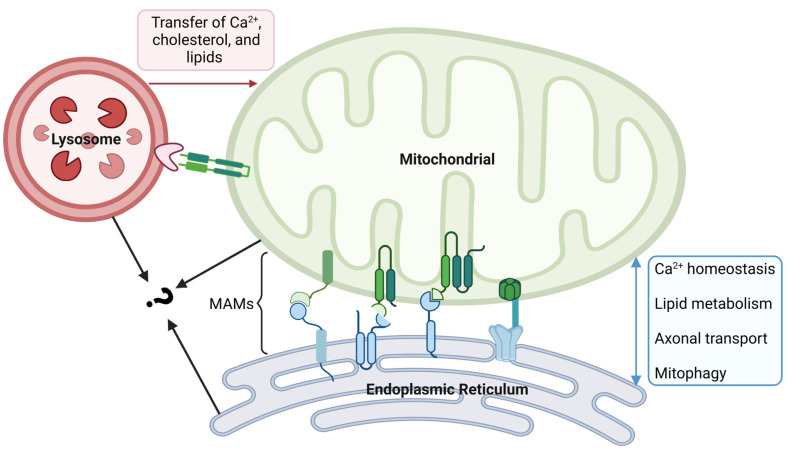
Crosstalk between mitochondrial and other organelles. The physical contact between mitochondria and the endoplasmic reticulum (ER) is made through mitochondria-associated membranes (MAMs). In MAMs, proteins with different roles are divided into four types. MAM is an important platform for Ca^2+^ exchange, lipid metabolism, axonal transport, and mitophagy. Crosstalk between mitochondria and lysosomes is established by inter-organelle membrane contact sites, which facilitate the transfer of Ca^2+^, cholesterol, and lipids from the lysosomes to mitochondria. Mitochondria and lysosomes in contact with each other can come into contact with the ER at the same time; however, the synergy between them remains unclear and needs further exploration. The figure was created using BioRender.com (https://biorender.com/, accessed on 20 December 2023).

Mitochondria are highly dynamic, and they work together with other organelles to coordinate different cellular mechanisms in space and time. The contacts between mitochondria and the ER play crucial roles in various cellular functions, including mitochondrial ATP production, biosynthetic processes, Ca^2+^ homeostasis, lipid metabolism, axonal transport, and mitophagy [103,104] (Figure 3). It is estimated that approximately 5–20% of the total mitochondrial surface is in intimate contact with the ER membrane, and these physical contacts between mitochondria and ER are called mitochondria-associated membranes (MAMs) [105]. Within the MAMs, proteins with different roles are categorized into four types, including tethering proteins, regulatory proteins, MAM biological function executive proteins, and ER-resident proteins [106]. They work together to maintain the structural and functional stability of the MAMs. Studies have shown that the pathogenesis of various neurodegenerative diseases, such as Alzheimer’s disease (AD), Parkinson’s disease, and Huntington’s disease, is closely related to the alterations of ER–mitochondria tethering and MAMs [104]. AMD, as the same type of disease, may have a similar pathological process. For example, amyloid β (Aβ) is the core component of amyloid plaques, and its formation is the key pathological process in AD. The production and regulation of Aβ was shown to be related to MAMs, and it has been found that Aβ can increase the number of ER–mitochondria contact points [107]. In dry AMD patients, Aβ was also found in drusen that contribute to local inflammation [108]. This evidence suggests that changes in MAMs may also play an important role in the development of AMD.

In addition, MAMs are important Ca^2+^ exchange platforms, providing a buffer zone for Ca^2+^ transfer between mitochondria and ER. When Ca^2+^ is released from the ER through IP3R or ryanodine receptors, it enters the mitochondria via the spatial relationship established by MAMs [109]. The Ca^2+^ influx in the MAM area can affect multiple aspects of mitochondrial function, and as consequence, modulate the synthesis of ROS [110]. Thus, the Ca^2+^ changes in MAMs may also be involved in the pathogenesis of AMD via ROS overproduction.

### 4.2. Mitochondria and Lysosomes

A key function of RPE cells is to maintain photoreceptor homeostasis, wherein they phagocytose and recycle old POS through lysosome-mediated autophagy. The disruption of the autophagy–lysosomal pathway increases susceptibility to RPE degeneration, which is one of the hallmarks of AMD. Thus, functional changes in lysosomes are deeply involved in the process of AMD. Although lysosomes have previously been considered static organelles that function in the disposal and recycling of cellular waste, the new discoveries confirm that they are in fact highly dynamic organelles. With the cooperation of other organelles, they adapt to changes in cell metabolism and cope with environmental stress. The crosstalk between lysosomes and mitochondria is established by inter-organelle membrane contact sites [111]. This intracellular communication is maintained by tethering proteins at an average distance of 10 nm between the mitochondria and lysosomes, with an average stable tethering time of 60 s [112]. Functionally, the contacts have been found to play diverse roles in metabolic signaling, the regulation of organelle dynamics, and additional cellular processes, including ROS production and mtDNA replication [113,114]. In detail, the transfer of Ca^2+^, cholesterol, and lipids from lysosomes to mitochondria at mitochondria–lysosome contact sites helps to maintain the dynamic equilibrium of metabolites (Figure 3). This kind of interaction occurs both under normal conditions as well as in response to cellular stress, and the dysregulation of the contacts has been shown to be associated with AMD. It is already known that the small GTPase Rab7 modulates the tethering and untethering dynamics of mitochondria and lysosomes [115]. Rab7 can be recruited to damaged mitochondria to participate in autophagy, which is critical for maintaining the homeostasis of mitochondria. A study observed the upregulation of Rab7 in the perinuclear space of RPE cells in a dry AMD mouse model, suggesting that changes in contacts may be involved in the disease process.

In addition, mitochondria and lysosomes that are in contact with each other can simultaneously contact the ER, indicating the contacts between organelles are not a simple point-to-point relationship [112]. The modulation of proteins at ER contacts may further influence the tethering and function of mitochondria–lysosome contacts [116].

## 5. Therapy

It has been shown in a study that autoantibodies can develop 3–15 years prior to the first clinical symptoms appear [117]. Therefore, the level of autoantibodies may provide clues for the early detection of diseases and the monitoring of disease progression. As the most important treatment for wet AMD, the effectiveness of the anti-VEGF treatment has been confirmed to be related to the levels of certain autoantibodies. In relevant clinical studies, changes in serum anti-retinal antibody levels coincided with the clinical outcomes of anti-angiogenic therapy, which could serve as markers of the efficacy of VEGF inhibitory treatments [118,119]. Although there are currently no clinical trial data available directly targeting autoantibodies as a therapeutic approach to manage AMD, drawing on the treatment methods of other autoimmune diseases, both the direct inactivation of specific antibodies and the indirect treatment of specific mitochondrial functions could be good choices.

ROS, a product primarily generated by mitochondria, is the instigator of oxidative stress in AMD. Therefore, targeting ROS has been deemed as an important strategy for the treatment of AMD. Studies have indicated that a range of dietary antioxidants, including multiple vitamins (vitamins A, C, E, and ß-carotene), minerals (zinc, copper, and selenium), as well as lutein and zeaxanthin could protect against oxidative injury. These supplements effectively quench the ROS activity, thus reducing the likelihood of developing AMD [120,121]. Enzyme antioxidants are another option for the treatment and prevention of AMD, acting as scavengers of ROS. Studies have shown that the overexpression or transduction of glutathione reductase, glutathione peroxidase, glutathione, and catalase in RPE cells could produce a protective effect against oxidative stress [122,123]. In addition, substances such as metformin and melatonin have been shown to play a therapeutic role in AMD treatment by affecting mitochondrial function to reduce H_2_O_2_-induced retinal oxidative stress [68,124].

Lipid deposition and drusen formation in Bruch’s membrane are the hallmarks of AMD. During this process, the dysregulation and accumulation of cholesterol contributes to the development of AMD by promoting inflammation and oxidation. Medications targeting lipid metabolism, such as desipramine, docosahexaenoic acid, apolipoprotein mimetics, and statins, are currently being considered as effective treatments for AMD [125]. As mitochondria are involved in cholesterol regulation, one of the therapeutic options for lipid metabolism intervention is to maintain normal lipid oxidation levels in RPE cells by preserving mitochondrial function [126]. In one study, RPE-specific mitochondrial transcription factor A (TFAM)-knockout mice exhibited an increased activation of the mammalian target of rapamycin (mTOR) pathway, which plays an important role in promoting lipid biosynthesis [125,127]. Conversely, inhibiting mTOR in the TFAM-knockout mice alleviates RPE pathologies. These results suggest that the preservation of the lipid oxidative pathway via the protection of mitochondrial function could be a promising way in AMD treatment.

Mitochondrial-derived peptides (MDPs) encoded by mtDNA can act as signals for the organism’s cytoprotecting and energy regulation. As another therapeutic option, it has already shown benefits in the treatment of AMD [128]. Well-known MDPs include humanin and small humanin-like peptides (SHLPs), both encoded by the 16S rRNA region of mtDNA. Humanin was found to rescue primary RPE cells from oxidative stress through enhanced mitochondrial biogenesis, and its variant humanin G could prevent Aβ-induced toxicity by restoring the mitochondrial membrane potential and calcium homeostasis, as well as reducing intracellular ROS levels [129,130]. SHLPs include SHLP 1-6, among which SHLP2 has been found to stabilize mitochondria in AMD and promote mitochondrial metabolism. Moreover, it has demonstrated protective effects against Aβ-induced toxicity in AMD RPE cybrid cells by preventing mitochondrial dysfunction [131]. These results suggest the role of MDPs as a candidate treatment for dry AMD, and they may potentially slow down the progression to advanced forms of the disease [128].

Gene therapy is a method that achieves the desired therapeutic effect by modifying defective DNA in recipient cells or tissues. Currently, gene therapy for AMD mainly focuses on the utilization of vector systems to express anti-angiogenic proteins that can block the VEGF pathway, especially in the context of wet AMD [132]. At the same time, gene therapy targeting mitochondria has also come to our attention. A study used gene therapy to directly boost mitochondrial function via the adeno-associated virus delivery of an optimized NADH–ubiquinone oxidoreductase (NDI1) gene, which provided robust benefits in multiple murine and cellular models of dry AMD [133]. In vitro, with the rescue of mitochondrial morphology and function, the cellular ROS levels and cell viability of primary RPE cells were improved. In vivo, the treatment reduced the ROS levels and improved ERG readings. These results provide us with a new strategy for treating dry AMD using gene therapy.

## 6. Conclusions

The presence of autoantibodies in the serum of AMD patients may contribute to the disease in two aspects. On the one hand, it could act as a predisposing factor that initiates the disease at the early stage. On the other hand, it may catalyze the disease progression at a late stage. For example, some autoantibodies can trigger the release of VEGF. Apart from their functions as biomarkers, these autoantibodies show the part that is likely to be primarily attacked during the disease. In this review, we focused on the pathogenesis associated with mitochondria and summarized the related therapeutic methods.

Mitochondria, as an important organelle involved in the regulation of both normal retinal activities and the pathological processes of AMD, function through multiple mechanisms. These include OxPhos, ROS production, Ca^2+^ storage, the maintenance of iron homeostasis, the regulation of cholesterol metabolism, and various other processes. Although ATP generation is thought to be the primary role of mitochondria, it is now known that mitochondria are multifunctional organelles, making it difficult to determine which functional changes have played a decisive role in advancing AMD. Therefore, further exploration is needed to clarify this issue and obtain a more comprehensive understanding. In addition, the communication between organelles is important for maintaining the physiological functions of cells. Although the complete interaction network remains unclear, there is growing evidence that a defective crosstalk between organelles is the underlying mechanism for the pathogenesis of many diseases, including cancer and a variety of neurodegenerative diseases. In AMD, how the abnormal crosstalk between organelles affects the internal environment and how this drives AMD progression may be the focus of future research.

Currently, therapies based on autoantibody profiling are being applied, which helps to develop more precise and personalized treatments. For example, therapies targeting mitochondrial autoantibodies have been widely used in many diseases. The treatment works by improving or disrupting mitochondrial function to achieve the desired effect. This kind of treatment strategy has also begun to rise in AMD, but the way of drug delivery and the cumulative effects still require further investigation and need to be paid attention to in future research. Furthermore, it must be acknowledged that we still do not know whether autoantibodies are a specific component of the AMD pathological process or simply markers for other factors involved in AMD pathology. This may make the effectiveness of therapies targeting autoantibodies uncertain.

## Figures and Tables

**Table 1:** Mitochondria-associated autoantigens in AMD patients.

Protein Name	MW (kDa)	Autoantibody-Related Mitochondrial Function	References
Alpha-enolase	46	ATP depletion and increase in intracellular Ca^2+^	[25]
Alpha-synuclein	14.5	Regulating iron homeostasis	[25,26]
Annexin V	35.9	Ca^2+^ binding	[26]
ATP synthase	56.6	ATP synthesis	[25]
Glial fibrillary acidic protein	52	ROS generation and Ca^2+^ release	[21,26]
Malate dehydrogenase	35.5	Marker of the mitochondrial matrix	[25]

## Data Availability

Not applicable.

## Abbreviations

Aβ	amyloid β
AD	Alzheimer’s disease
Alpha-enolase	α-enolase
Alpha-synuclein	α-syn
AMD	age-related macular degeneration
APO	apolipoprotein
ATP	adenosine triphosphate
CNV	choroidal neovascularization
DNA	deoxyribonucleic acid
ER	endoplasmic reticulum
ETC	electron transport chain
Fe-S	iron–sulfur
GA	geographic atrophy
GFAP	glial fibrillary acid protein
H_2_O_2_	hydrogen peroxide
IMM	inner mitochondrial membrane
IMS	intermembrane space
MAMs	mitochondria-associated membranes
MDPs	mitochondria-derived peptides
mtDNA	mitochondrial DNA
mTOR	mammalian target of rapamycin
mPTP	mitochondrial permeability transition pore
NDI1	NADH–ubiquinone oxidoreductase
NRFs	nuclear respiratory factors
O_2_^−^	superoxide anion radical
OH^·^	hydroxyl radicals
OxPhos	oxidative phosphorylation
PGC	peroxisome proliferator-activated receptor γ coactivator
PR	photoreceptors
RNA	ribonucleic acid
RO^·^	alkoxy radical
ROO^·^	peroxyl radical
ROS	reactive oxygen species
RPE	retinal pigment epithelium
SHLPs	small humanin-like peptides
TCA	tricarboxylic acid cycle
TF	transferrin
TFAM	mitochondrial transcription factor A
TFR	transferrin receptor
TSPO	transporters
VEGF	vascular endothelial growth factor
